# Polysaccharide hydrogels for skin wound healing

**DOI:** 10.1016/j.heliyon.2024.e35014

**Published:** 2024-07-22

**Authors:** Meifen Liu, Jiman Jin, Xiqiang Zhong, Liangle Liu, Chengxuan Tang, Limei Cai

**Affiliations:** The Third Affiliated Hospital of Wenzhou Medical University, Wenzhou, 325200, China

**Keywords:** Polysaccharide, Regenerative medicine, Skin wound, Hydrogel, Tissue repairing

## Abstract

Advances in the development and utilization of polysaccharide materials are highly promising, offering prominent applications in the field of tissue engineering for addressing diverse clinical needs, including wound healing, bone regeneration, cartilage repair, and treatment of conditions such as arthritis. Novel polysaccharide materials are popular owing to their inherent stability, biocompatibility, and repeatability. This review presents an overview of the biomedical applications of natural polysaccharide hydrogels and their derivatives. Herein, we discuss the latest advancements in the fabrication, physicochemical properties, and biomedical applications of polysaccharide-based hydrogels, including chitosan, hyaluronic acid, alginate, and cellulose. Various processing techniques applicable to polysaccharide materials are explored, such as the transformation of polysaccharide hydrogels into electrospun nanofibers, microneedles, microspheres, and nanogels. Furthermore, the use of polysaccharide hydrogels in the context of wound-healing applications, including hemostatic effects, antimicrobial activities, anti-inflammatory properties, and promotion of angiogenesis, is presented. Finally, we address the challenges encountered in the development of polysaccharide hydrogels and outline the potential prospects in this evolving field.

## Graphical abstract

**Figure:**
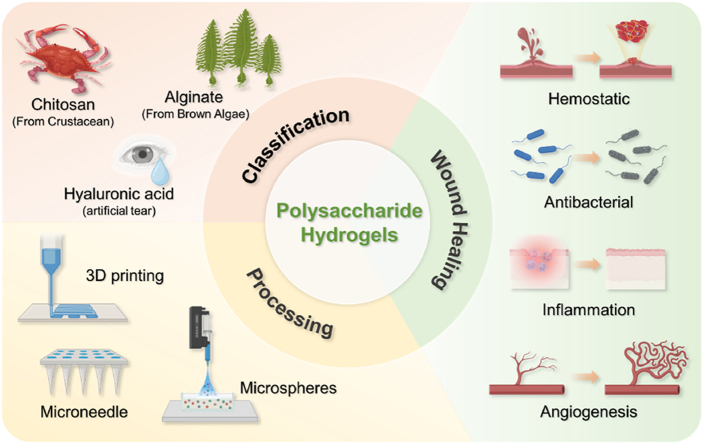

## 1 Introduction

Skin is essential for numerous functions, including sensing external stimuli and shielding the body tissues from external injuries [1]. However, owing to its constant exposure to the external environment, skin tissue is highly susceptible to damage. Although various skin injuries can heal naturally, with the tissue returning to its original state over time, abnormal wound healing can be induced by factors such as excessive inflammation [[2], [3], [4], [5]], burns [6,7], extensive skin tissue loss [[8], [9], [10]], infections [5], and diabetes [[11], [12], [13]]. In addition to concealing wounds, wound dressings function as instructive templates that direct cell reorganization. Efficient wound dressings should exhibit exceptional tissue compatibility and degradability without inducing toxicity or inflammation [[14], [15], [16]]. In addition, they should encourage cell adhesion, proliferation, and differentiation; stimulate the formation of blood vessels; and promote the synthesis of connective tissue [10,17,18]. Furthermore, they should offer protection against bacterial infections, maintain a moist environment at the wound site, and absorb wound exudate [19,20].

Hydrogels are considered an ideal choice for dressings owing to their exceptional efficiency in drug delivery and their ability to create a moist environment that expedites the healing process [8,9,[21], [22], [23]]. Moreover, hydrogels possess remarkable swelling capabilities, enabling them to absorb excess exudates, and their porosity can be adjusted to accommodate various cells, drugs, and nanotrace elements [[24], [25], [26]]. In addition, with a three-dimensional (3D) structure resembling that of the extracellular matrix (ECM), hydrogels offer the advantage of preservation of biomolecule and cell activitity [27,28]. Therefore, the demand for natural hydrogels with favorable attributes is growing.

The exploration of natural polysaccharides as hydrogels is compelling because of its abundance in nature, enhanced biocompatibility, and biodegradability. In addition to being abundant in nature, natural polysaccharides exhibit excellent biocompatibility and biodegradability [29,30]. Furthermore, the various active hydroxyl, carboxyl, and amino groups present in the monosaccharide units provide opportunities for derivatization [31,32]. This facilitates diverse crosslinking processes for hydrogel formation and imparts unique characteristics that enable interactions with living organisms [[33], [34], [35]]. Polysaccharide-based hydrogels can sustain local concentrations of bioactive substances over extended periods through suitable release mechanisms including diffusion, swelling, chemical factors, and control of specific environmental stimuli. This allows the precise and controlled release of drugs or nutrients, underscoring the inherent advantages of polysaccharide-based hydrogels as carriers for encapsulating such substances [36,37]. Moreover, polysaccharide-based hydrogels exhibit impressive mechanical properties and biological activity, rendering them potent for cell culture and tissue regeneration [[38], [39], [40]]. As research on polysaccharide-based hydrogels sourced from natural sources continues, their exceptional biological activity is gaining broader recognition within the scientific community.

Herein, we offer a comprehensive overview of current research on polysaccharide hydrogels for wound healing. We start by discussing the skin wound-healing process and the parameters used to assess it. Subsequently, we present common polysaccharide materials, such as chitosan (CS), hyaluronic acid (HA), and alginate, and the various hydrogel processing techniques, including electrostatic spinning, microneedles, and microspheres. This review comprehensively explores the role of polysaccharide hydrogels in wound treatment, highlighting their hemostatic, antibacterial, anti-inflammatory, and proangiogenic properties. The primary objective of this review was to provide guidelines for the formulation of polysaccharide hydrogels tailored for wound healing while also delving into the significant roles of such materials in the therapeutic process.

## 2 Skin injuries

The skin, being the largest multilayered organ of the human body, serves as a crucial barrier against pathogenic invasions. However, when the entire epidermis is severely compromised (such as through surgical procedures, burns, or skin cancer), the skin experiences significant blood loss and a diminished protective function. Particularly, microbial infection at the wound site markedly prolongs the healing process. In the context of skin repair, various cellular pathways are activated to restore skin integrity and hemostasis, in conjunction with endothelial and immune cells, growth factors, and hormones [41,42].

### 2.1 Skin injury types

Skin injuries are generally categorized into two main types based on the healing time: acute and chronic. Acute wounds resulting from surgical procedures or traumatic events typically follow a normal healing process and involve tissue regeneration. Platelets, fibroblasts, and microvascular cells collaborate to facilitate wound repair. In contrast, chronic wounds, including burn injuries and diabetic ulcers, remain in the inflammatory phase for an extended period [13,43].

Burn dressings maintain a moist environment on the burned skin, absorb exudates, and assist in scab removal. The healing outcomes of burn injuries depend on the severity of the injury. Superficial burns typically heal within two weeks and result in minimal scarring. In deeper burns, the healing process commences at the edges of the wound rather than at the center, as rapid closure of the wound is crucial.

Diabetic foot ulcers represent a significant medical concern in diabetic individuals [11]. The normal healing process is disrupted in diabetic wounds, leading to prolonged inflammation and microcirculatory deficiencies. This heightened and protracted inflammatory phase is primarily driven by the infiltration and sustained presence of dysfunctional macrophages and neutrophils at the wound site [12]. Immune cells recruited to the wound site during inflammation generate excessive reactive oxygen species (ROS). Additionally, owing to compromised immune responses, diabetic wounds are vulnerable to bacterial infections, resulting in increased ROS production and intensified inflammatory reactions. In contrast, diabetic wounds exhibit impaired cellular behavior, including compromised fibroblast proliferation and migration, reduced recruitment of endothelial progenitor cells, cellular damage, and apoptosis.

### 2.2 Skin injury repair

**Fig. 1:**
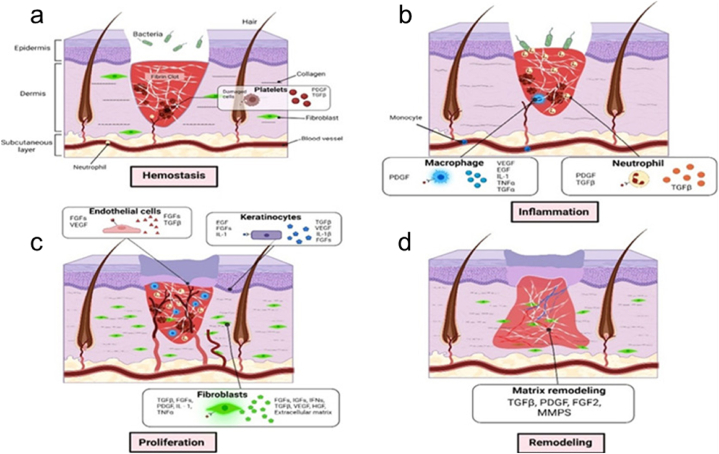
The stages of wound healing. (a) hemostasis, (b) inflammation, (c) proliferation, and (d) remodeling [46]. Adapted reprinted with permission from Ref. [46], License Number: 5,807,930,870,062. *Copyright © 2023 Elsevier B.V. All rights reserved*.

A normal skin structure typically consists of the epidermal and dermal layers. The epidermal layer directly interfaces with the external environment and is composed primarily of the stratum corneum and germinative layer. It has various functions, including prevention of tissue fluid efflux, resistance to friction, and defense against infection [[44], [45], [46]]. The dermal layer, conversely, is composed of dense connective tissue and is divided into the papillary and reticular layers arranged from superficial to deep. Wound regeneration involves four sequential processes: hemostasis, inflammation, proliferation, and remodeling (Fig. 1a–d).

Hemostasis is the initial response of the body to wounds. Following injury, the blood vessels promptly constrict themselves to minimize bleeding. Subsequently, the platelets aggregate to seal the ruptured vessel walls. Thrombin facilitates the formation of a fibrin network that aggregates platelets into solid clots. Ultimately, a thrombus forms at the wound site [41].

During inflammation, neutrophils and monocytes differentiate into macrophages. This inflammatory response aids in the clearance of foreign substances and involves chemotactic secretion to attract cells. The origin of wound-site macrophages is linked to monocyte differentiation and local tissue residence. In the stages of inflammation, proinflammatory macrophages (M1) produce ROS and proinflammatory cytokines that exert antimicrobial effects. Driven by various signals, M1 macrophages polarize into anti-inflammatory macrophages (M2), facilitating their transition into the proliferation phase [47].

The proliferation phase encompasses wound filling and re-epithelialization. During this phase, fibroblasts migrate to the injury site, proliferate, and secrete proteases and ECM components to form granulation tissue [48]. Basal cells around the damaged tissue proliferate and migrate to form epithelial cells. When epithelial cells cover the entire wound surface, re-epithelialization is complete. Specifically, angiogenesis is a critical process during the proliferation phase.

The remodeling phase represents the final stage. At this stage, collagen is replaced gradually by type I collagen in the granulation tissue [44]. The ECM in the granulation tissue undergoes reorganization and repositioning, improving its distribution and enhancing tissue tensile strength. In addition, hair and sweat glands may undergo regeneration.

## 3 Classification and crosslinking mechanisms of polysaccharides

Understanding the classification and cross-linking mechanisms of polysaccharides is crucial to mastering their biomedical applications, especially in polysaccharide hydrogels.Polysaccharides such as CS, HA, and sodium alginate are biocompatible and biodegradable, and thus are widely used in drug delivery, tissue engineering, and wound healing [49]. The cross-linking mechanism is divided into physical and chemical cross-linking, which can regulate the mechanical strength, swelling behavior and degradation rate of hydrogels [36]. By understanding these mechanisms, researchers can optimize the properties of polysaccharide-based hydrogels to enhance their functionality and expand their applications in biomedical fields.

### 3.1 Classification

**Fig. 2:**
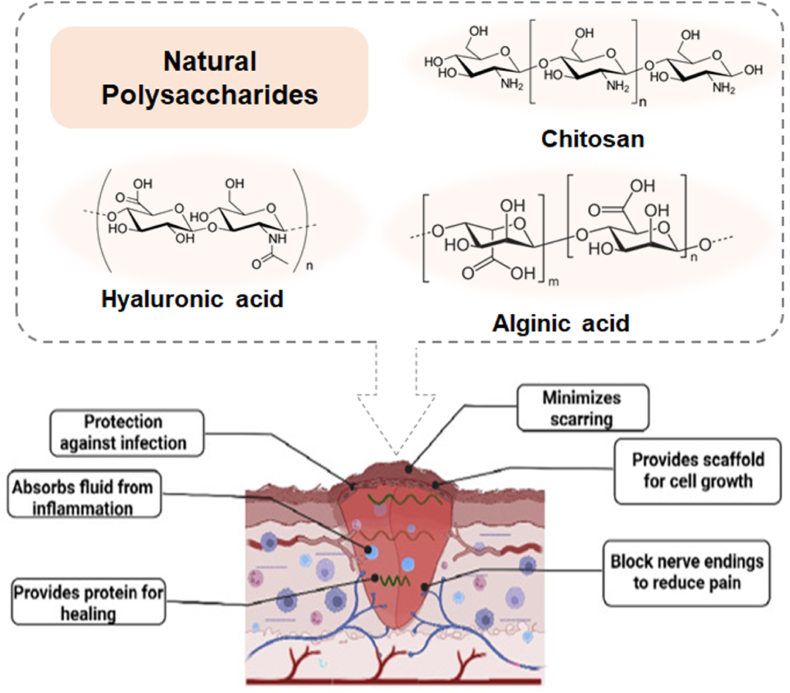
Wound healing action of polysaccharide-based hydrogels [38]. Adapted reprinted with permission from Ref. [38], License Number: 5,807,940,123,195. *Copyright © 2022 Elsevier B.V. All rights reserved*.

Polysaccharides are generally categorized according to their source (animal, plant, algal, and microbial), structure (linear or branched), and charge (anionic, cationic, or neutral) [50,51]. The biological activity of polysaccharides varies according to their specific chemical properties [39,46]. Therefore, the chemical structure strongly influences the function and application of polysaccharides. Chitosan, sodium alginate, HA, and their derivatives, are vital polysaccharides in wound healing applications due to their biocompatibility and unique properties (Fig. 2). Chitosan, with its antimicrobial and hemostatic properties, accelerates healing and prevents infections. Sodium alginate, derived from brown seaweed, forms hydrogels that are highly absorbent and suitable for exudative wounds, providing a moist environment that promotes healing. Hyaluronic acid, a natural glycosaminoglycan, enhances tissue hydration, cell migration, and angiogenesis, supporting re-epithelialization and tissue regeneration. These polysaccharides are used in advanced wound dressings, leveraging their bioactive effects to improve wound care outcomes.

#### 3.1.1 Chitosan

Chitosan exhibits a range of distinct properties, including biodegradability, biocompatibility, antimicrobial activity, hemostatic capacity, and cell affinity [52]. It has widespread clinical applications in wound treatment and delivery of bioactive substances [53,54]. In addition, it contains free amino groups and is a cationic polymer of natural origin. Owing to its cationic nature, it readily interacts with negatively charged bacterial membranes, leading to the extrusion of cellular proteins and other components, thereby exerting significant antimicrobial effects and inhibiting bacterial growth within wounds [55,56]. Consequently, CS is used extensively as a reparative material for wound dressings.

Chitosan exhibits pH-responsive characteristics because alkaline solutions cause degradation of its chain. Chemical modifications such as the introduction of chemical moieties, including alkyl and carboxymethyl groups, into the CS structure, can enhance its solubility under different pH conditions [57]. Among such modifications, carboxymethyl CS, a key CS derivative, demonstrates improved biocompatibility and biodegradability compared with unmodified CS [14]. Additionally, grafting hydrophobic alkyl chains onto a chitosan (CS) backbone can enhance its hemostatic and anti-infective properties, owing to the strong hydrophobic interactions between the alkyl chains and the membranes of red blood cells (RBCs), platelets, and bacteria [[58], [59], [60]]. Xinchen Du et al. developed an anti-infective shape-memory hemostatic sponge with enhanced pro-coagulant and hemostatic capabilities. They engineered a microchannelled alkylated chitosan sponge using 3D printing, which can facilitate in situ tissue regeneration for noncompressible hemorrhages in both civilian and battlefield settings [61]. Similarly, CS/polyethylene oxide/kaolin nanofiber membranes have been fabricated using electrospinning technology to create a superior structure for wound healing [62].

#### 3.1.2 Hyaluronic acid (HA)

Hyaluronic acid is composed of repeating disaccharide units comprising glucuronic acid and N-acetyl-d-glucosamine [63], and its molecular weight ranges from 10^4^ to 10^7^. It contains numerous hydroxyl and carboxyl groups, which provide abundant active sites [64]. It can form hydrogen bonds with water and other hydrophilic substances and undergo chemical reactions to create new polymers with various compounds. Huinan Suo et al. developed a HA composite hydrogel, incorporating an antimicrobial peptide as a cross-linking agent through Schiff's base formation. This hydrogel exhibited an acidity-triggered release of AMP in the pathological environment of bacteria-infected wounds. Their work provides an efficient strategy for fabricating antibiotic-free hydrogel-based biomaterials for bacteria-infected wounds [65]. Hyaluronic acid is found naturally in the human body and is distributed widely in connective tissues, neural tissues, bodily fluids, and epithelial cells, providing exceptional hydration capabilities.

Hyaluronic acid has various essential physiological roles owing to its unique molecular structure and physicochemical properties [[66], [67], [68]]. These include joint lubrication, regulation of vascular wall permeability, and facilitation of the diffusion and operation of proteins, water, and electrolytes. As an essential component of ECM, it enhances early inflammation, supports cell infiltration, accelerates the development of granulation tissue matrices, and enhances tissue remodeling [69,70]. For instance, Liu et al. reported an electrospun thioether-grafted hyaluronic acid nanofiber, which could spontaneously form a nanofibrous hydrogel in situ on the wound bed, synergistically modulating the inflammatory microenvironment and accelerating chronic diabetic wound healing [71]. Hyaluronic acid exhibits high hydrophilicity, biodegradability, and biocompatibility, rendering it suitable for wound dressings.

#### 3.1.3 Alginate

Alginate is extracted primarily from the cytoplasm of brown algae. It contains hydroxyl groups that can interact with divalent metal ions, such as Ca^2+^ and Mg^2+^, and metal oxides [51]. Its efficiency in wound repair is attributed to its excellent hydrophilicity, biocompatibility, biodegradability, and low toxicity. Chen et al. demonstrated that a polyphenol-based supramolecular injectable hydrogel (PBSIH) can be rapidly formed within 15 s by mixing sodium alginate and gallic acid solutions, driven by hydrogen bonding and hydrophobic interactions [72]. The PBSIH demonstrates excellent cytocompatibility, antibacterial, and antioxidant properties, which augment the healing of infected wounds by inhibiting bacterial infection and reducing inflammation after an 11-day treatment period. A multifunctional oxidized alginate (OA)/polydeoxyribonucleotide (PDRN) hydrogel system was similarly developed via ionic crosslinking and Schiff base reaction between OA and PDRN. Biocompatibility assessment of the PDRN-loaded OA hydrogels revealed a significant enhancement in cell viability in human dermal fibroblast cells [73]. Alginate hydrogels absorb substantial amounts of wound exudates, protect wounds from microbial intrusion, and provide a moist environment [74,75]. Calcium alginate was mixed with AgNPs formulation to prepare a composite sponge, which exhibited significant antimicrobial activity against gram-positive, gram-negative, and fungi, and the sponge outperformed other commercially available silver nanoparticle-loaded AgNPs-loaded wound dressings for wound healing [76]. Furthermore, alginate hydrogels do not adhere to wound tissues, and their removal does not induce secondary damage to the wound surface.

#### 3.1.4 Cellulose

Cellulose is the primary structural component of the plant cell wall. The presence of numerous hydroxyl groups along the cellulose main chain results in poor dispersibility in common solvents owing to intermolecular and intramolecular hydrogen bonding [77]. Natural cellulose is typically improved via physical and chemical modification or enzymatic hydrolysis to enhance its efficacy. Cellulose derivatives, such as hydroxyethyl cellulose, carboxymethyl cellulose, and hydroxypropyl methylcellulose, have been widely used as scaffold materials in hydrogels. The incorporation of highly absorbent carboxymethyl cellulose generates soft gel-like cellulose hydrogels that transition to hydrogels upon contact with the wound exudate, being highly effective for moderate to heavily exuding wounds [51]. Cellulose hydrogels are typically mixed with other polymer materials and are chemically modified to enhance wound healing because of their low mechanical strength.

#### 3.1.5 Dextran

Dextran is a biopolymer consisting of α-1,6-linked glucose monomers with α-1,3-branching. Dextran is known for its facile production and biocompatibility, rendering it widely applicable in various fields [42]. Its properties contribute to maintaining wound cleanliness, eliminating exudates, metabolic waste, and other unfavorable factors, ensuring a conducive environment for healing. Moreover, it can promote angiogenesis, protect the epidermis from ischemic injury, stimulate granulation tissue formation, enhance the healing process, and promote collagen deposition for tissue remodeling [78]. Dextran hydrogels can be engineered to possess specific structural characteristics and encapsulate vascular growth factors to promote skin repair. Oxidized and acetylated dextran derivatives have been explored to accelerate wound repair and maximize their therapeutic potential.

#### 3.1.6 Starch

Starch is a high-molecular-weight polysaccharide composed of numerous glucose units. The ratio of amylose to amylopectin affects its crystallinity, molecular order, and gelatinization. Starch is affordable, nontoxic, biodegradable, and biocompatible. However, the extended chain structure of starch is vulnerable to hydrolysis by gastric acids and enzymes, limiting its application in wound healing [51,79]. To overcome this challenge, natural starch can be modified via physical treatments, chemical modifications, or enzymatic hydrolysis. Furthermore, for advanced applications that require enhanced performance, starch can be modified by forming physically linked networks with other polymers.

#### 3.1.7 Other polysaccharides

Pectin is an anionic polymer extracted from plants, comprising galacturonic acid, rhamnose, xylose, fructose, and other components. It typically consists of three polysaccharide domains: homogalacturonic acid, high rhamnose, and rhamnogalacturonan. Pectin-based wound dressings facilitate exudate removal, maintain the acidic wound environment, and inhibit bacterial growth [80,81].

Carrageenan is a high-molecular-weight natural polysaccharide extracted from red seaweeds [82]. The gel viscosity of carrageenan hydrogels is determined by the reaction type and temperature, as well as the molecular weight and concentration of carrageenan. As the temperature increases, the interactions between the large molecular chains lead to an increased viscosity of carrageenan hydrogels. When the temperature decreases, the kappa-form of carrageenan is influenced by positively charged ions, transitioning from a spiral rearrangement and aggregation to the formation of ionotropic and thermally reversible hydrogels [83,84]. Kappa-carrageenan has the potential to generate thermally reversible gels, offering various possibilities for application in the food and pharmaceutical sectors. Carrageenan hydrogels mimic the natural microenvironment, allowing for improved interactions between cells and tissues. Moreover, the sulfate groups of carrageenan promote blood clotting and immune function, making it suitable for biological applications.

### 3.2 Crosslinking mechanisms

**Fig. 3:**
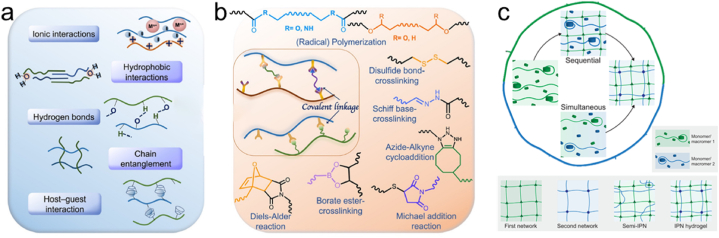
Schematic representation of (a) physical or (b) chemical cross-linking methods for designing polysaccharide hydrogels [35]. Adapted reprinted with permission from Ref. [35], License Number: 5,790,741,112,426. *Copyright © 2021 Elsevier Ltd. All rights reserved.* (c) Network structures of fully interpenetrating polymer network (IPN) and semi-interpenetrating polymer network (semi-IPN) hydrogels [85]. Adapted reprinted with permission from Ref. [85], License Number: 5,791,811,331,290. *Copyright © 2020 Elsevier Ltd. All rights reserved.*

The synthetic routes for polysaccharide hydrogels can be categorized into physical and chemical crosslinking methods (Fig. 3a and b) [85]. Physical crosslinking is based on physical interactions, such as hydrogen bonding, ionic interactions, crystallization, and hydrophobic interactions. Moreover, it does not require additional crosslinking agents to create hydrogels. Physical crosslinking can establish a 3D network via methods such as repetitive freezing and thawing, as well as pH adjustment, without using chemical crosslinkers.

In contrast, chemical crosslinking techniques include enzyme-mediated crosslinking, functional group-mediated chemical reactions, and radiation technology. The morphology and biomechanical properties of polysaccharide-based hydrogels prepared via chemical crosslinking can be enhanced using chemical crosslinkers. Some studies have investigated the construction of double-network hydrogels by combining multiple hydrogel networks. The following sections provide detailed insights into these methods.

#### 3.2.1 Physical crosslinking

Physically crosslinked hydrogels involve various forces, including ionic interactions, hydrogen bonding, hydrophobic interactions, polymer chain entanglement, and host-guest interactions. These forces lead to the formation of semi-permanent junctions within the polymer network, strengthening the overall structure and enhancing the ability of the hydrogel to interact with and retain water molecules.

The ionic interactions in alginate, CS, and cellulose derivatives are essential for the formation of physically crosslinked hydrogels. Divalent metal cations such as biocompatible calcium salts can interact with saccharide blocks, creating ionic bridges that physically connect the alginate chains and form hydrogels [86]. Tang et al. used ionotropic regulation techniques to prepare CA/CS hydrogel spheres with good adsorption capacity and reusability [87]. In contrast, Duffy et al. prepared CS microgels via gelation in an iron solution [88].

Hydrogen bonds play a synergistic role in the functions of biological tissues. These dynamic and reversible chemical bonds can covalently link and detach, thereby regulating the strength of the hydrogels, playing an important role in self-healing hydrogels. Zhao et al. used hydrogen-bonding interactions to self-assemble sodium alginate into a porous matrix, creating a layer-by-layer interpenetrating network structure hydrogel (PAMSA) [89]. PAMSA demonstrated an impressive self-recovery rate; its mechanical properties could be fully restored to their original levels after self-recovery.

Hydrogels are prone to breakage in the presence of water, leading to reduced mechanical performance and increased energy consumption, therefore limiting their applications. To address this issue, researchers have introduced hydrophobic moieties into the hydrophilic polymer chains. For instance, pentenyl groups can be generated as hydrophobic chains through ultraviolet radiation, inducing strong hydrophobic interactions that maintain the hydrogel in a “non-swollen” state [90]. Hydrophobic interactions effectively dissipate energy in the form of sacrificial bonds. Zhang et al. developed double-network self-healing hydrogels using CS and poly (stearyl methacrylate) (PSBMA) [91]. After the disruption of the hydrogel, the ionic crosslinks between the CS networks and micelles in the PSBMA network were also disrupted. The hydrogel was cut, and the detached hexadecyl trimethylammonium chloride and SBMA alkyl groups formed new micelles. Furthermore, the aminoglucose units of CS were coordinately linked to the citrate ions.

**Fig. 4:**
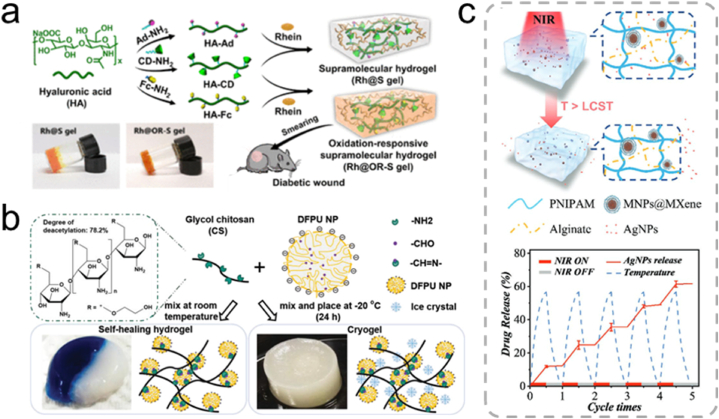
(a) Synthesis of supramolecular hydrogels based on ferrocene and β-cyclodextrin subject-guest recognition, and a schematic illustration of their application in chronic wound healing [92]. Adapted reprinted with permission from Ref. [92]. *Copyright © 2020, American Chemical Society.* (b) Synthesis of self-healing hydrogels via Schiff base bonding between CS and polyurethane [94]. Adapted reprinted with permission from Ref. [94], based on CC BY License. *Copyright © 2019 The Authors. Published by WILEY‐VCH Verlag GmbH & Co. KGaA, Weinheim.* (c) Drug-release behavior of PNIPAM-alginate-based dual network hydrogels [95]. Adapted reprinted with permission from Ref. [95], License Number: 5,791,830,555,629. *Copyright © 2021 Wiley‐VCH GmbH.*

Host-guest interactions are established through supramolecular recognition and binding. They play a pivotal role in regulating the degree of crosslinking as well as controlling the strength and self-healing properties of hydrogels. The development of self-healing hydrogels utilizing host-guest interactions, particularly β-cyclodextrin (β-CD), has garnered significant attention. β-CD possesses a distinct hydrophobic internal cavity and an outer hydrophilic wall, rendering it promising for encapsulating various molecules to form host-guest layers. Modification of HA with ferrocene and β-CD using the host-guest macromonomer approach afforded a supramolecular hydrogel with high sensitivity to oxidative microenvironments, being suitable for treating inflammatory wounds (Fig. 4a) [92]. The dynamic properties of reversibly crosslinked host-guest supramolecular hydrogels play an essential role in regulating the growth behavior of encapsulated cells within a 3D network [93].

#### 3.2.2 Chemical crosslinking

Chemical hydrogels are typically formed via chemical crosslinking by reacting crosslinking agents with polysaccharide derivatives. Polysaccharide derivatives can be triggered by light, electricity, or heat to create crosslinked networks that are held together by covalent bonds. Compared to physical hydrogels, chemical hydrogels tend to exhibit greater mechanical strength [85]. The most common method for chemical crosslinking is free-radical polymerization, which typically requires the use of chemical initiators to initiate the reaction. The formation of dynamic covalent bonds can also establish hydrogel crosslinked structures. These include classic click chemistry (azide-alkyne cycloaddition), Diels–Alder reactions, Michael addition reactions, and the formation of Schiff bases, disulfide bonds, borate esters, and coordination bonds. Such stimuli-responsive hydrogels have found application in various fields as carriers for local delivery of therapeutic agents, offering controlled drug release.

Imine compounds, also known as Schiff bases, are named after the German chemist Hugo Schiff. They contain an iminomethylene group (-RC=N-) and are typically formed by the condensation of primary amines with active carbonyl compounds. The imine bond resulting from Schiff base reactions is a reversible dynamic covalent bond that can break and reassemble without external stimuli. Lin et al. developed a self-healing hydrogel using polyurethane and CS to create a Schiff base-crosslinked hydrogel known as CS-PU, exhibiting self-healing properties at room temperature (Fig. 4b) [94].

Oxime bonds, formed by the condensation of oxime and carbonyl groups, are dynamic and reversible covalent bonds similar to imine bonds. Oxime bonds are sensitive to pH and temperature; under weakly acidic conditions, they break and regenerate rapidly, offering control of sol-gel transitions by adjusting the pH. Kim et al. introduced oxime bonds to produce self-healing hydrogels by combining oxidized HA with ethylene glycol CS (GC) via Schiff base bonds, followed by reaction with adipic dihydrazide to form oxime bonds [96]. The resulting hydrogel self-healed within 10 min and was suitable as a cell-loaded structure for cartilage regeneration.

The formation of borate-ester bonds via a reaction between boronic acid and diols is a reversible interaction. The multifunctional hydrogel formed from 3-aminophenyl boronic acid and alginate exhibited self-healing, injectability, and pH-responsive behavior under alkaline conditions. However, alginates do not react with boronic acid compounds. Therefore, functional groups that can react with boronic acid must be introduced into the alginate chain. Wu et al. grafted 3-aminophenyl boronic acid onto sodium alginate to introduce a boronic acid salt [97]. Boronic acid salts bond with the hydroxyl groups on the polyethylene oxide surface, forming borate ester bonds that exhibit excellent self-healing properties.

#### 3.2.3 Double-network hydrogels

Double-network hydrogels, a category of polymer blends, are formed via continuous interpenetration of different types of crosslinked or linear polymers into a network structure. They can be further classified as interpenetrating polymer networks (IPN) and semi-interpenetrating polymer networks (semi-IPN) hydrogels (Fig. 3c). In IPN hydrogels, all the polymer components are crosslinked, forming interpenetrating networks, whereas semi-IPN hydrogels consist of both crosslinked and non-crosslinked polymers within the network structure.

During hydrogel preparation, achieving the desired mechanical strength can be challenging when natural polysaccharides are the only components. Conversely, synthetic polymers, whose mechanical properties are easily modulated, typically lack biodegradability and optimal cell adhesion. Double-network hydrogels combine natural and synthetic polysaccharides and can harness the intrinsic properties of polysaccharides while delivering a high mechanical performance. These hydrogels exhibit significantly enhanced mechanical strength compared to single-network gels. For example, Yang et al. proposed a light- and magneto-responsive double-network hydrogel composed of poly (N-isopropyl acrylamide) (PNIPAM) and sodium alginate (Fig. 4c) [95]. PNIPAM is a temperature-responsive polymer that contracts at high temperatures, whereas the alginate-based, safe, and nontoxic semi-interpenetrating network enhanced the mechanical properties of the hydrogel system. This innovative hydrogel exhibited great potential for applications in drug delivery and treatment of deep chronic wounds.

## 4 Polymeric hydrogel processing

The fabrication of polysaccharide-based hydrogels using advanced technologies such as 3D printing, electrospinning, microspheres and microneedles is important for biomedical applications. Three-dimensional printing technology can facilitate tissue engineering and regenerative medicine by precisely fabricating customized hydrogel scaffolds according to the different needs of patients. Electrospinning technology generates nanofibrous mats that mimic extracellular matrix to promote cell adhesion and proliferation, thereby facilitating wound healing and drug delivery applications. Microspheres help in controlled drug release and targeted therapy, while microneedles enable minimally invasive drug delivery and localized therapy. These innovative processing methods enhance the functionality and therapeutic efficacy of polysaccharide-based hydrogels, highlighting their importance in advancing biomedical science and improving patient care.

### 4.1 Three-dimensional printing

3D printing involves the automated deposition of ink in layers to create specific components. The most common 3D printing techniques include inkjet-, extrusion-, and light-based methods [98]. Natural and synthetic polymer hydrogels are typically used in biomanufacturing owing to their ability to provide a 3D and cell-friendly environment with mechanical integrity. Synthetic polymers, such as polycaprolactone (PCL), polylactic acid, and poly (lactic-*co*-glycolic acid), are commonly used in 3D printing owing to their high printability and mechanical strength. However, their use is limited by the elevated printing temperatures and the need for organic solvents.

**Fig. 5:**
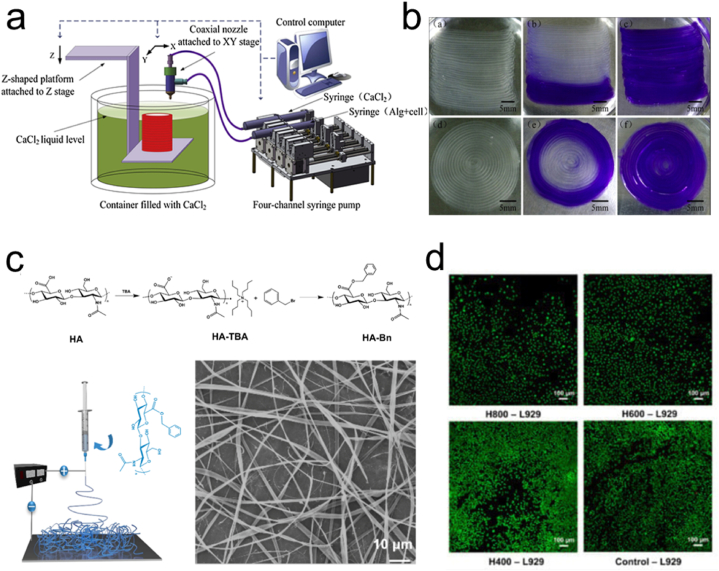
(a) Experimental setup of a coaxial nozzle-assisted 3D bioprinting system. (b) 2D-printed alginate structures with built-in microchannels [100]. Adapted reprinted with permission from Ref. [100], License Number: 5,791,941,172,214. *Copyright © 2015 Elsevier Ltd. All rights reserved.* (c) SEM images and schematic diagram of HA-Bn Synthetic. (d) Live/dead staining [102]. Adapted reprinted with permission from Ref. [102], based on CC BY License. *Copyright ©* 2023 by *the authors. Licensee MDPI, Basel, Switzerland.*

Alginate, gelatin, and CS offer enhanced biocompatibility and reduced cellular toxicity, with alginate hydrogels being widely used for 3D bioprinting [99]. Gao et al. developed a novel 3D bioprinting method based on hollow CA filaments using coaxial nozzles (Fig. 5a and b) [100]. Wu et al. 3D printed human corneal epithelial cells/collagen/gelatin/alginate hydrogels and obtained cell-loaded tissue constructs with controlled degradation [101]. However, the development of hydrogels that are suitable for 3D printing and meet biological requirements and printing fidelity remains a significant challenge.

Current research is focused on the design and application of 3D printing technology for medical and tissue engineering, using various types of biomaterials and advanced manufacturing techniques. For example, Remaggi et al. extracted derivatives from a subspecies of *Lactobacillus bulgaricus* to prepare self-crosslinking 3D-printed alginate/HA hydrogels, which showed promise for regenerative medical applications, particularly in promoting skin wound healing [103]. Han et al. prepared HA derivative (HA-Bn) nanofibers by benzylation reaction using HA as a raw material (Fig. 5c and d). By adjusting the spinning parameters, electrostatically spun membranes with various fiber diameters were obtained. These fibrous membranes have good biocompatibility. HA-Bn electrostatically spun membranes can promote the regeneration of injured skin tissues and bind with drugs for therapeutic effects, providing a powerful way to develop therapeutic and regenerative biomaterials. In addition, Zhang et al. introduced a novel CS ink 3D printing strategy, achieving rapid printing of high-strength CS hydrogels through temperature-induced gelation, with potential applications in tissue engineering and drug delivery [104]. Such studies collectively demonstrate the potential applications of 3D printing technology in the field of medicine, driving innovations in tissue regeneration and therapies using various biomaterials and improved printing techniques.

### 4.2 Electrospinning

**Fig. 6:**
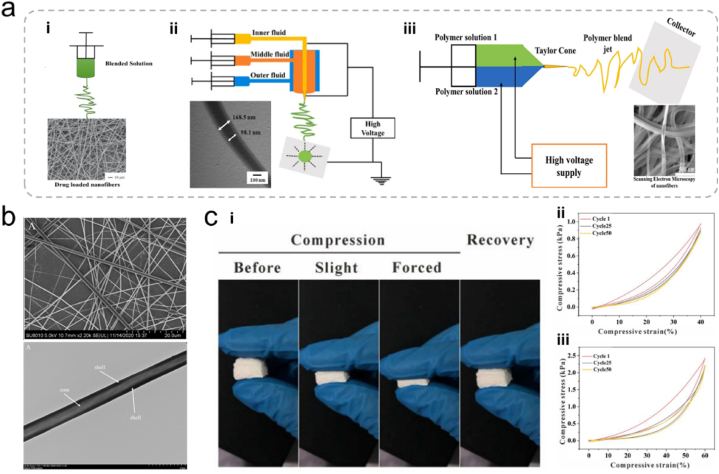
(a) Schematic of nanofibers prepared by the (i) blended, (ii) coaxial electrospinning, and (iii) side-by-side electrospinning techniques [105]. Adapted reprinted with permission from Ref. [105], License Number: 5,792,251,387,042. *Copyright © 2023 Elsevier B.V. All rights reserved.* (b) Polyvinyl alcohol/pectin shell core fibers prepared by coaxial electrostatic spinning [106]. Adapted reprinted with permission from Ref. [106], License Number: 5,792,260,783,519. *Copyright © 2022 Elsevier Ltd. All rights reserved.* (c) (i) Photographs of a 3D CS/PVA nanofiber sponge recovering after different levels of compression. (ii) Compression fatigue test of a 3D CS/PVA nanofiber foam conducted in 50 cycles [107]. Adapted reprinted with permission from Ref. [107], License Number: 5,792,261,017,593. *Copyright © 2019 Elsevier Ltd. All rights reserved.*

Electrospinning is used to fabricate nanofibers from various polymers. It has become popular for producing micro-/nanofibers with diverse morphologies, patterns, and functions. Electrospinning is an electrohydrodynamic process that involves the charging of liquid droplets to produce a jet that stretches and forms fibers. The electrospinning setup typically includes an injection pump, spinneret, high-voltage generator, and collector. During electrospinning, the liquid is extruded from the spinneret, forming spherical liquid droplets that become conical owing to electrostatic repulsion, known as the “Taylor cone” [105]. The liquid jet ejected from the Taylor cone undergoes an intense whipping motion and is stretched to finer diameters, resulting in solidified nanofibers that are deposited on the collector (Fig. 6a) [105]. Various processing parameters, including the applied voltage, liquid viscosity, conductivity, and distance between the spinneret tip and the collector, significantly influence the formation and dimensions of electrospun nanofibers.

Polysaccharide electrospun nanofibers offer unique biological properties and hold great promise for wound-healing applications. Compared with synthetic polymers, naturally derived polymers exhibit high biocompatibility and biodegradability, rendering them advantageous bioink materials. The development of functional biomaterials involves various natural polymers because they can support cellular processes.

Particularly, single-nozzle electrospinning has been extensively studied and is commonly employed to create single-layer nanofiber membranes. Co-axial electrospinning requires multiple components to be soluble in the same solvent. This process is straightforward and offers flexibility in terms of solvent selection, making it an attractive option. Jirofti et al. employed co-axial electrospinning to fabricate nanofibers that encapsulate curcumin within a matrix of CS, polyethylene oxide, and collagen (CS/PEO/Col) [108]. These newly developed electrospun nanofibers loaded with curcumin effectively controlled bacterial growth.

Although the straightforward blending of drugs with materials is a reproducible method for preparing drug-loaded nanofibers, it typically results in a burst release of the drug from the nanofiber surface owing to the material's high surface area. This burst release of the drugs is opposing the desired sustained release over an extended period. Furthermore, some delicate drugs, such as proteins, can be unstable in organic solvents, making nanofibers less suitable for wound healing applications [105,109]. To address these challenges, researchers have developed co-axial electrospinning techniques.

Co-axial electrospinning utilizes concentric needle structures to spray two components simultaneously. This prevents interference between the components and allows for the encapsulation of drugs or active substances within the core of the nanofibers. In this approach, the drugs are inside the core and gradually dissolve or are released through the hydrophilic pores within the shell, resulting in a slower release. For example, Xu et al. successfully prepared core-shell nanofibers using Eudragit S100 and polyvinyl alcohol (PVA)/pectin via co-axial electrospinning (Fig. 6b) [106]. Compared to electrospun fibers prepared using single-nozzle electrospinning, *Lactobacillus rhamnosus* encapsulated in core-shell fibers exhibited higher thermal stability. Furthermore, core-shell fiber encapsulation of probiotics enhanced their tolerance to harsh gastrointestinal conditions.

Single-layer nanofiber membranes prepared by co-axial or single-nozzle electrospinning offer relatively simple properties and may not achieve the complex functions required for advanced wound healing. To address this issue, researchers have focused on developing wound dressings from polysaccharide nanofiber membranes with multilayered spatial structures. For instance, Zhang et al. made significant progress in this area by designing sponges with a hierarchical structure that includes 3D nanofiber scaffolds. This innovative approach enhances interactions with blood cells, ultimately accelerating hemostasis (Fig. 6c) [107]. The fine-tuning of the structure afforded 3D nanofiber sponges with favorable properties for wound healing, such as good elasticity, high permeability, and efficient fluid absorption. These multilayered structures are promising for addressing the demands of wound healing and enhancing the effectiveness of wound dressings.

Certain polysaccharides may exhibit inadequate electrospinnability because of their naturally high molecular weights and charges. These properties can result in poor mechanical performance and limited water resistance. In contrast, synthetic polymers typically exhibit excellent electrospinning abilities and superior mechanical properties. To overcome this challenge, researchers have employed electrospinning to fabricate nanofibers from high molecular-weight synthetic polymers. These synthetic nanofibers can subsequently be coated with polysaccharides to create polysaccharide-coated nanofibers. This approach offers various advantages as it combines the outstanding mechanical properties of synthetic polymers with the hygroscopicity, biocompatibility, and wound-healing properties of polysaccharides. For example, Dodero et al. used electrospinning to create multilayer nanofiber membranes comprising layers of polycaprolactone and physically crosslinked alginate embedded in zinc oxide nanoparticles [110]. In this study, polycaprolactone acted as a co-spinning agent for alginate and did not disrupt the unique nanofiber structure of the membrane. The outer layer of polycaprolactone enhanced the liquid-repellent properties of the sample, whereas the alginate layer promoted tissue regeneration. This approach demonstrates the potential of combining synthetic and polysaccharide materials to create nanofibers with improved performances for various applications, including wound healing.

### 4.3 Microneedles

Microneedles, which consist of an array of miniature needles, have gained significant attention as innovative drug delivery systems owing to their minimally invasive nature, ease of use, localized controllability, and versatile cargo-loading capabilities [16,111]. Although microneedles were initially designed for transdermal drug delivery, recent years have revealed numerous emerging applications, including their role in promoting wound healing and various tissue regeneration processes.

The microneedle structure allows for the loading of various pharmaceutical agents. The microscale needle tips can breach the physical barrier, enabling sustained drug release and enhanced drug delivery efficiency. Compared to conventional subcutaneous injections, microneedles offer painless and non-invasive drug administration, which significantly improves patient compliance [112,113]. Furthermore, owing to their needle array configuration, microneedles can provide mechanical stimulation and induce collagen deposition and reorganization, ultimately reducing scarring. Various materials, including metals, silicon, silicon dioxide, ceramics, and hydrogels, have been used in the fabrication of microneedles to satisfy the diverse requirements.

Natural polysaccharide hydrogel microneedles such as HA, CS, and pullulan are characterized by relatively poor mechanical properties and short degradation durations [16]. Such features render them suitable for applications requiring rapid drug release, such as early-stage wound healing for infection control. Furthermore, natural polysaccharide hydrogel microneedles exhibit adjustable polymer network density that can be designed to control drug release based on the size of the drug molecules.

Micromolding is a common employed technique for fabricating microneedles, involving the use of a negative mold with needle cavities to load the materials [112]. Once the materials are filled and cured, the microneedles can be demolded from the negative mold, completing the microneedle fabrication. This method is suitable for preparing various polysaccharide hydrogel microneedles. Furthermore, depending on the specific characteristics of the materials, various curing and shaping methods can be used.

The micromolding technology is known for its simplicity, cost-effectiveness, and high reproducibility [114]. Different materials and cargos can be sequentially added to the same mold, allowing the creation of composite microneedles. This approach can be used to obtain structures with complex release kinetics and functions, including core-shell and stacked structures, as mentioned earlier. However, at the microscale level, where the surface tension becomes dominant, filling the mold cavities with the gels under the influence of gravity is extremely challenging. To overcome surface tension, various methods have been employed to facilitate mold filling, such as vacuum assistance, centrifugation, stamping, electrospinning coating, atomization spraying, and permeation. Furthermore, the micromolding technology have limitations in creating intricate structures, such as bonding structures with hooks or hollow structures. Additionally, natural polysaccharide hydrogels such as alginate are soft and fragile, and the demolding stress can potentially damage the final structure.

**Fig. 7:**
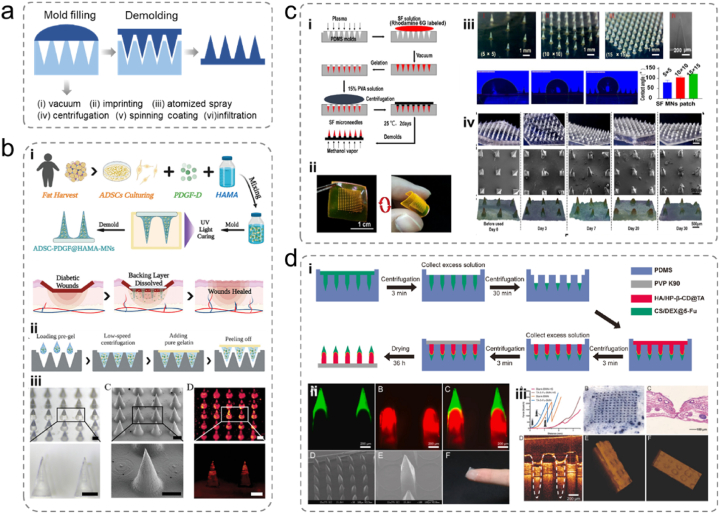
(a) Typical micro-injection molding processes and six injection-filling methods. (b) (i) Schematic of the MN system loaded with ADSCs and PDGF-D for diabetic wound therapy. (ii) Schematic of the fabrication process of the MNs arrays. (iii) Optical image, Scanning Electron Microscopy (SEM) photo, and fluorescence image of ADSC-PDGF@HAMA-MNs wrapped with fluorescent probe labelling [115]. Adapted reprinted with permission from Ref. [115], License Number: 5,792,350,949,641. *Copyright © 2022 Wiley‐VCH GmbH.* (c) (i) Fabrication of silk fibroin microneedle patches, (ii) microneedle patch morphology, (iii) contact angle, (iv) morphology of microneedle patches before and after insertion into the rabbit ear scar [116]. Adapted reprinted with permission from Ref. [116], *Copyright © 2022 The Authors. Published by American Chemical Society. This publication is licensed under CC-BY-NC-ND 4.0.* (d) (i) Schematic of the bilayer dissolving microneedle (BMN) fabrication process, (ii) confocal micrographs and SEM images of fluorescent BMNs, (iii) photograph of skin surface after insertion of BMN [117]. Adapted reprinted with permission from Ref. [117], based on CC-BY–NC–ND License. *Copyright © 2021 The Authors.*

Polysaccharide microneedles are already used in skin tissue treatment and wound monitoring, thereby aiding wound management and improving treatment outcomes. In contrast to the complex cell-loading processes described earlier. Xu et al. produced cell-laden microneedles to heal diabetic ulcers. They used basic micromolding methods and mixed ADSCs and a platelet-derived growth factor (PDGF-D) directly with methacrylated HA (HAMA) to form needle tips with a gelatin backing layer (Fig. 7a and b) [115]. PDGF-D facilitated ADSC proliferation, differentiation, and migration, whereas more than 90 % of the needle tip cells maintained their vitality. The produced microneedles exhibited a thick granulation tissue in the PDGF-D- and ADSC-laden microneedle groups, the stratum corneum was the most intact, the collagen deposition was organized and dense, and neovascularization was the best.

Microneedles offer a painless and effective way of delivering drugs to deeper tissues. They are beneficial for disrupting the protective membranes of bacteria, overcoming biofilm resistance, and improving treatment outcomes. Deng et al. proposed an antimicrobial microneedle system inspired by the teeth of hagfish designed for wound healing in cases of infection [118]. This system was created by mixing silk fibroin with antimicrobial zinc oxide nanoparticles (ZNPs) through micromolding. The needle structures varied within the same patch, with the shorter central needles being placed vertically and the longer peripheral needles being inclined inwards, resembling the teeth of hagfish. This structural design provided a shearing force that facilitated wound closure and accelerated wound healing. Bioinspired microneedles loaded with ZNPs significantly enhanced the healing process and suppressed infections. Liang et al. used micromolding and developed a US-activated microneedle system composed of HA dispersed in CuO_2_/TiO_2_ [119]. Upon ultrasound activation, CuO_2_ released Cu^2+^ ions, which were internalized by bacteria and subsequently transformed into toxic ROS free radicals that eradicate bacteria. Ultrasound waves were also effectively absorbed by TiO_2_ and converted into heat, causing thermal damage to bacteria. In a mouse wound infection model of multidrug-resistant *Staphylococcus aureus*, the proposed microneedle system demonstrated superior antimicrobial activity. It reduced inflammation and promoted collagen alignment in wound tissues, highlighting its potential for infection control and wound healing.

Burn injuries can lead to the formation of hypertrophic scars (HS) and keloids, which are associated with severe complications. For example, Zhang et al. proposed a non-drug microneedle system for HS treatment consisting of silk fibroin (SF) needles and PVA backing layer manufactured via micromolding (Fig. 7c) [116]. In vivo experiments revealed that the SF microneedles significantly improved scar appearance, whereas histological analysis revealed decreased collagen deposition and reduced cell counts in the scar regions. Protein imprinting confirmed that the microneedles downregulated the expression of fibrosis markers TGF-β1 and α-SMA. Furthermore, the physical interventions induced by the microneedles reduced mechanical communication in scar tissue. Yang et al. introduced a double-layer soluble microneedle system loaded with triamcinolone acetonide (TA) and 5-fluorouracil (5-Fu), both commonly used for HS treatment (Fig. 7d) [117]. The needle tip is composed of CS and dextran to encapsulate 5-Fu for sustained release. In contrast, the needle shaft is made of HA integrated with TA using hydroxypropyl-β-cyclodextrin for rapid TA release. The backing layer is constructed from polyvinylpyrrolidone and manufactured using a multistep micromolding process. After five treatments with the TA-5-Fu-loaded microneedles, scar redness was reduced, the skin surface was smoother, and significant reductions in the thickness of the epidermis and dermis were observed. The fibroblast alignment in the scar tissue became more regular, indicating the potential of this microneedle system for HS treatment.

### 4.4 Microspheres

**Fig. 8:**
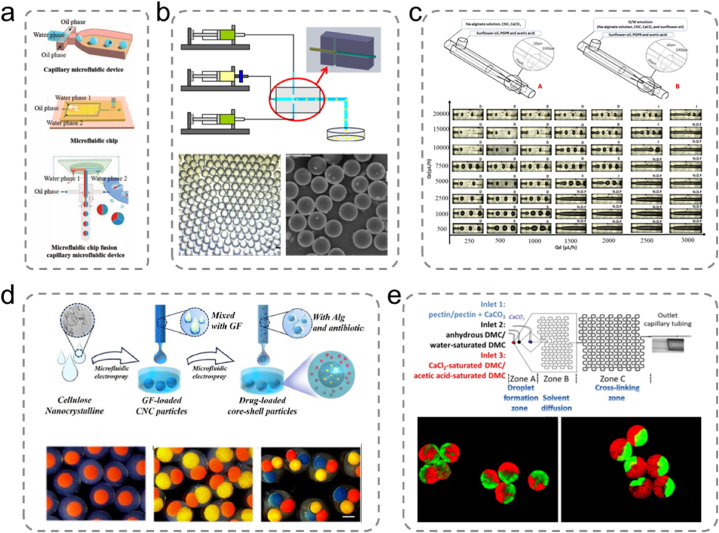
(a) Devices used for the preparation of microfluidic hydrogel microspheres [28]. Adapted reprinted with permission from Ref. [28], License Number: 5,792,500,948,298. *Copyright © 2021 Wiley‐VCH GmbH.* (b) Schematic of the coaxial microfluidic device for the preparation of chitosan (CS) microspheres and optical and scanning electron micrographs of CS microspheres [121]. Adapted reprinted with permission from Ref. [121], License Number: 5,792,510,975,707. *Copyright © 2012 Elsevier B.V. All rights reserved.* (c) Schematic of a co-flow glass microfluidic device for the production of alginate microspheres and the process of droplet formation under different continuous and dispersed flow rate conditions [122]. Adapted reprinted with permission from Ref. [122], License Number: 5,792,520,088,612. *Copyright © 2021 Elsevier Ltd. All rights reserved.* (d) Generation of multicompartmental core-shell particles consisting of cellulose nanocrystals and alginate containing growth factors and antibiotics, respectively. Optical microscopy images of monodisperse multicompartmental core-shell particles with one, two, and three nuclei [123]. Adapted reprinted with permission from Ref. [123], License Number: 5,792,530,184,633. *Copyright © 2021 Elsevier Ltd. All rights reserved.* (e) Schematic of a microfluidic flow focusing chip for pectin hydrogel microparticle formation. Fluorescence image of Janus hydrogel microparticles [124]. Adapted reprinted with permission from Ref. [124], License Number: 5,792,530,968,511. *Copyright © 2014 Elsevier Ltd. All rights reserved.*

Microspheres are micron-sized spherical particles composed of one or more polymer phases in which drugs and other components can be dispersed or dissolved [120]. Polysaccharide hydrogel microspheres are advanced functional materials offering controlled drug and cell delivery for a wide range of biomedical applications. Various methods are available for processing microspheres depending on the material properties and intended applications. Common methods for producing polysaccharide hydrogel microspheres include emulsification, microfluidics, and microfluidic electrospraying (Fig. 8a) [28]. These methods enable the fabrication of tailored microspheres with specific characteristics depending on the application.

Physical crosslinking relies on the unique physical properties of materials, whereas chemical crosslinking involves chemical bonding. Shi et al. harnessed the distinctive in situ thermal gelation behavior of alkaline CS solutions to produce physically crosslinked CS microspheres [125]. Similarly, Wang et al. fabricated konjac/CS microspheres using the emulsion crosslinking method [126]. Chemical solvents that stimulate emulsification can also be used in certain cases. Consequently, additional processing steps are necessary to minimize adverse effects on downstream cell viability. Notably, microspheres prepared via the emulsion method typically exhibit nonuniform sizes, which can be challenging for downstream applications that require precise size distributions.

Spray drying is a method that entails mixing drugs and gel materials into a solution, followed by spraying the solution into an inert heated airflow [120,127]. During the process, the solvent evaporates, and the polymer precipitates into spherical structures containing the encapsulated drug. The approach offers various advantages, including minimal pollution, high solution precision, good sealing properties, and low shear forces. Zhang et al. employed CS as a carrier and herbaceous extracts as raw materials to establish drug microspheres that protect the gastric mucosa. They utilized the spray-drying method, which demonstrated favorable in vitro sustained-release effects [128]. Similarly, Mouez et al. encapsulated verapamil hydrochloride in CS microspheres via spray drying, resulting in excellent encapsulation efficiency and release performance [129].

The self-assembly method relies on weak forces, such as hydrogen bonds, intermolecular covalent bonds, and electrostatic interactions, to spontaneously form regular polymer structures. This production process is simple and involves mild reaction conditions. It is suitable for both hydrophilic and hydrophobic drugs. However, improving the size and dispersity of the microspheres is challenging. Chen et al. prepared silk fibroin nanospheres loaded with the anticancer drug paclitaxel using the self-assembly method [130]. In contrast, Cheng et al. employed the self-assembly method to establish silk fibroin microspheres [131].

Conversely, the microfluidic technology is highly effective in controlling multiphase fluids precisely. In a microfluidic emulsion system, oil and water converge within geometrically designed microchannels, leading to droplet formation [28,33,132]. These droplets are subsequently crosslinked by physical or chemical means, resulting in the establishment of microfluidic hydrogel microspheres. The method yields microspheres with remarkable size uniformity and extremely narrow size distributions. Compared to other methods of microsphere preparation, microfluidic approaches offer several advantages, such as rapid microsphere preparation and microspheres with high monodispersity and adjustable sizes owing to the controllable flow rates. Furthermore, improvement of the microfluidic devices can lead to hydrogel microspheres with various complex functionalities. Considering such advantages, manufacturing monodisperse engineered microspheres with controlled sizes, specific functionalities, and various forms is possible, rendering them promising for biomedicine applications.

The fundamental principle behind microfluidic microsphere preparation relies on the shear forces generated at the interface of the oil and water surfaces, aided by surfactants [133]. Moreover, control of the fluid displacement during microsphere formation by altering the ratio of the shear force between the fluids can lead to the formation of size- and shape-controllable hydrogel microspheres. These fluid dynamic principles enhance the efficiency and controllability of microfluidic hydrogel microsphere preparation.

For the production of microfluidic hydrogel microspheres, two types of devices are usually employed: glass capillary microfluidics and microfluidic chips. Capillary microfluidic devices are straightforward to fabricate, durable, and are typically assembled using an injection needle, transfer tubing, and collector. These devices manipulate liquids by harnessing the capillary effect, which is governed by the interplay between the surface chemical properties, surface tension, and geometric shape of the liquid. Following the formation of water-in-oil (O/W) or oil-in-water (W/O) single emulsion droplets, crosslinking is initiated by exposure to light, pH adjustments, heating, cooling, or chemical additives, resulting in the production of hydrogel microspheres. In addition, the microfluidic electrospray technology is an efficient and straightforward approach for microsphere fabrication involving a microfluidic electrospray device, which primarily consists of capillaries. Zhao et al. used a coaxial capillary microfluidic device to prepare monodisperse CS microspheres with enhanced autofluorescence and smaller diameters using a solvent extraction curing method in combination with chemical crosslinking (Fig. 8b) [121]. Meirelles et al. described a method for creating alginate hydrogel microspheres filled with emulsions using a microcapillary device (Fig. 8c) [122]. A continuous aqueous phase containing sodium alginate, cellulose nanocrystals, and calcium carbonate was initially prepared to form an O/W emulsion. Subsequently, the O/W emulsion was introduced into a continuous phase containing sunflower oil within the microfluidic device to produce an O/W/O emulsion. Internal gelation facilitated the solidification of the aqueous phase, thereby promoting the development of the alginate network.

Chen et al. utilized this method and introduced a mixture of cellulose nanocrystals and sodium alginate into a microfluidic electrospray setup, resulting in the formation of core-shell microspheres within a calcium chloride solution (Fig. 8d) [123]. They focused on the practical implications of these microspheres for wound healing; the alginate shell demonstrated the capacity to carry antibiotics and enable rapid release, owing to its inherently large pores, contributing to early-stage inflammation management during wound healing. As the outer layer degraded, the cellulose nanocrystals in the core hydrogel facilitated the gradual release of the loaded growth factors via the small pores within the robust gel network. This phenomenon can be attributed to the presence of rigid CNCs, inducing a “nano-locking effect,” promoting cell migration and proliferation in the later stages of wound healing. Lei et al. employed the microfluidic electrospray technology to generate biohybrid microspheres composed of agarose and HA for use in localized growth factor delivery in chronic wound healing [134]. Agarose, which is known for its high water-absorbing capacity, creates a moist wound environment that is conducive to healing. Liu et al. proposed a novel porous microcapsule using a coaxial microfluidic electrospray technique to encapsulate β-cells for diabetes treatment [135]. The porous hydrogel shell is composed of a sodium alginate and polyethylene oxide (PEO) mixture. It employs PEO as the porogen because it does not undergo crosslinking during the alginate gelation process. The internal liquid core consists of a β-cell solution dispersed in carboxymethyl cellulose sodium. Liu et al. achieved the formation of uniform microcapsule structures by employing microfluidic technology. The liquid core within these microcapsules provides a 3D culture environment for the enclosed β-cells, while the porous shell offers protection against immune rejection during the transplantation process. Furthermore, the porous structure of sodium alginate allows the exchange of small molecules required for β-cell viability, resulting in robust cell activity and functionality after encapsulation.

The microfluidic chip technology provides higher resolution and greater flexibility in channel design. Microfluidic chips exhibit biocompatibility, optical transparency, and breathability. They harness the unique properties of microfluids, such as rapid heat conduction, surface tension, capillary action, diffusion, and laminar flow effects, thereby facilitating fluid control and rapid responses. Microfabrication of microfluidic chips efficiently creates microchannels with specific internal structures and various small high-density microstructures. This enables the combination of operational units and scalable integration. For example, Marquis et al. combined the phenomenon of gelation on a chip with the self-assembly induced by water diffusion (Fig. 8e) [124]. This approach successfully produced complex-shaped pectin hydrogel microspheres with spherical, mushroom-like, annular, and elliptical morphologies. Compared with capillary devices, microfluidic chips offer advantages in terms of automation, efficiency, and miniaturization. However, they exhibit limitations in terms of reusability, device maintainability, and higher fabrication costs.

### 4.5 Nanogels

Nanogels are microscale hydrogels with dimensions ranging from a few nanometers to micrometers. They are typically referred to as hydrogel nanoparticles because they combine the advantages of hydrogels and nanoparticles. Nanogels exhibit high water retention, renewability, biodegradability, and biocompatibility [136]. The primary advantage of microgels over bulk hydrogels is their faster response to external stimuli (e.g., temperature, light, and pH), which is attributed to their smaller size. Furthermore, both nanogels and microgels can serve as minimally invasive drug delivery systems when used as drug carriers. Notably, the self-assembly of colloidal particles into nanogels provides numerous opportunities for applications in nanotechnology.

**Fig. 9:**
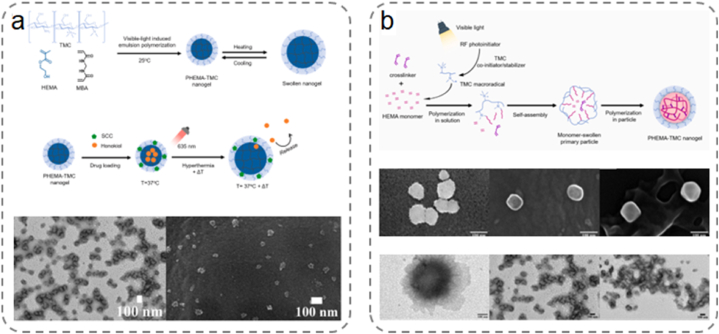
(a) Synthesis of PHEMA-TMC nanogels and their thermal response behavior. Transmission Electron Microscopy (TEM) and Scanning Electron Microscopy (SEM) images of a nanogel with phosphotungstic acid staining [137]. Adapted reprinted with permission from Ref. [137], License Number: 5,792,540,546,011. *Copyright © 2023 Elsevier B.V. All rights reserved.* (b) Schematic representation of visible light induced surfactant-free emulsion polymerization on TMC using amino compounds as initiation system. Scanning electron microscopy images of the nanogels and TEM images after phosphotungstic acid staining [138]. Adapted reprinted with permission from Ref. [138], License Number: 5,794,870,766,326. *Copyright © 2022 Elsevier Ltd. All rights reserved.*

Free-radical polymerization generates polymer particles of different sizes in various dispersion systems. The most common systems for synthesizing nanogels include emulsions, miniemulsions, microemulsions, and precipitation. Among these, precipitation polymerization offers the advantage of not requiring surfactants, rendering it the preferred method for manufacturing nanogels for in vivo applications. Lekjinda et al. employed a simple visible-light-initiated, surfactant-free emulsion polymerization to synthesize multi-stimuli-responsive biocompatible nanogels (Fig. 9a) [137]. The nanogels were composed of a crosslinked poly (2-hydroxyethyl methacrylate) (PHEMA) core and a trimethyl CS (TMC) periphery, serving as multi-stimuli-responsive nanocarriers (Fig. 9b) [138]. Recently, Lekjinda et al. synthesized environment-friendly PHEMA-TMC nanogels functionalized with TMC via surfactant-free emulsion photopolymerization. Leite et al. introduced smart starch-poly (N-isopropylacrylamide) microgels using a nanoprecipitation approach. The addition of surfactants during the microgel manufacturing process introduced starch nanoparticles in the continuous network [139].

The formation of nanogels via enzymatic catalysis has distinct advantages, including selectivity, substrate specificity, environmental friendliness, and mild reaction conditions. Enzymes play a crucial role in catalyzing the crosslinking of polymer chains and converting monomers into polymers suitable for nanogel construction. They also react with the nanogel precursors, facilitating their self-assembly into nanogels. Bocharova et al. harnessed the biocatalytic capabilities of multicopper oxidase laccase as both catalyst and template to grow monodisperse hydrogel nanoparticles with tunable sizes [140]. The nanoparticles were formed in a dilute polymer solution of alginate through crosslinking between alginate and iron (III) cations generated by enzymatic action.

## 5 Wound healing

The escalating commercial demand and rising incidence of wound injuries underscore the necessity for effective wound management. As a result, researchers have directed their efforts towards the advancement of wound care technologies [15]. Clinical practice requires wound dressings with multifunctional properties, all aimed at accelerating wound healing. These properties include hemostatic properties, antimicrobial capabilities, anti-inflammatory effects, and the ability to promote angiogenesis. Due to the great potential of natural polysaccharides in wound healing, researchers are actively developing a variety of functional polysaccharide hydrogels to address the clinical importance of wound healing.

### 5.1 Hemostasis

Hemorrhagic complications typically lead to high morbidity and mortality rates among civilians and military personnel with injuries. Effective hemostasis is of paramount clinical significance for prevention, surgery, and emergency medical care. Uncontrolled or profuse bleeding from irregular wounds is a major cause of traumatic death in situations such as wars or accidents. The principles and mechanisms of hemostasis have been comprehensively described in previous publications [141]. In clinical applications, effective hemostatic products for arterial bleeding include gauze and bandages. Traditional gauze is primarily composed of cellulose, exhibiting excellent biocompatibility, biodegradability, antimicrobial properties, and cost-effectiveness. However, traditional hemostatic gauzes lack the necessary in vivo degradation characteristics, making them unsuitable for surgical intervention. Therefore, the development of emerging wound dressings with superior hemostatic properties is of paramount importance for life-saving efforts.

The inherent characteristics of natural polysaccharides, such as high swelling and adsorption capabilities, nonallergenic nature, and biocompatibility, render them suitable for hemostatic applications. Moreover, they can undergo in vivo or ex vivo biodegradation into products that can be eliminated from the body with or without metabolic transformation. Recently, polysaccharide-based hydrogels have gained prominence in the design of hemostatic dressings because of their excellent biocompatibility and biodegradability [84]. Polysaccharides, such as CS, cellulose, alginate, starch, dextran, and HA, serve as core materials for such hemostatic dressings. Their inherent physicochemical properties, similarity to the skin tissue, compatibility with blood, and coagulation play crucial roles in their effectiveness.

**Fig. 10:**
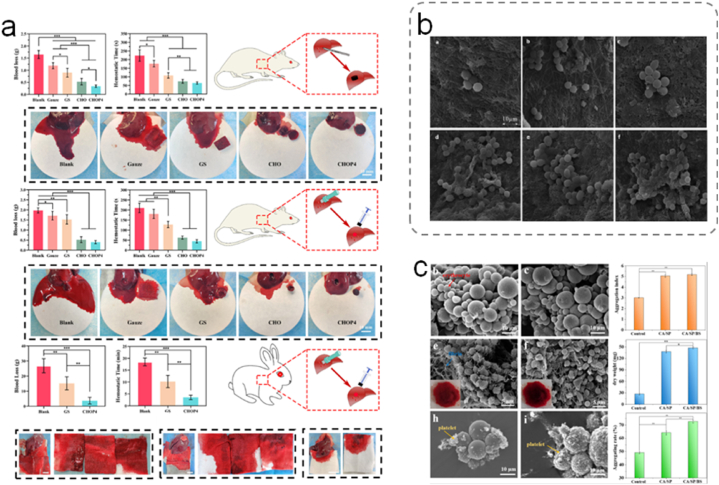
(a) CHOP cryogels prevent bleeding by promoting blood coagulation through physical compression of the bleeding site, aggregation and activation of blood cells, active adsorption of fibrinogen, activation of coagulation factors, and promotion of thrombin production [143]. Adapted reprinted with permission from Ref. [143], License Number: 5,795,341,277,224. *Copyright © 2022 Elsevier B.V. All rights reserved.* (b) KC and CMC hemostatic membranes. SEM micrographs of red blood cells adhered to the nanofibers after 2 h of incubation in blood [145]. Adapted reprinted with permission from Ref. [145], License Number: 5,795,351,140,548. *Copyright © 2023 Published by Elsevier B.V.* (c) Alginate, silk fibroin peptide, and *Bletilla striata* polysaccharide composite microspheres for hemostasis [146]. Adapted reprinted with permission from Ref. [146], License Number: 5,795,360,021,322. Copyright © 2022 Published by Elsevier B.V.

Various forms of CS have been investigated as hemostatic materials. Hou et al. co-polymerized GC methacrylate and ε-polylysine acrylamide at −20 °C to prepare dual-functional cryogel wound dressings, combining the hemostatic properties of CS with the high antimicrobial efficiency of antimicrobial peptides [142]. The cryogels exhibited a higher clotting rate than gauze or gelatin sponges. Furthermore, the incorporation of EPL resulted in an increased positive charge, leading to high coagulation rates in GC-EPL cryogels. Recently, the same team developed cryogels using oxidized dextran as a biocompatible crosslinker. They combined the excellent hemostatic properties of CS and human-like collagen with the introduction of polydopamine nanoparticles to enhance the shape-recovery rate of the cryogels, further enhancing their hemostatic performance (Fig. 10a) [143]. Zhao et al. designed a hydrogel composed of modified carboxymethyl CS and oxidized dextran with tannic acid doping into a hydrogen bond network [144]. The prepared hydrogel exhibited significant platelet aggregation and a reduced blood clotting index. The hydrogel rapidly adhered to and sealed wounds, demonstrating excellent hemostatic effects in vivo. Salmasi et al. prepared a kappa-carrageenan-carboxymethyl CS hemostatic membrane via electrospinning (Fig. 10b) [145]. Electrospinning increased the specific surface area of the membrane, therefore enhancing contact with blood. The negatively charged kappa-carrageenan sulfate groups activated factors XII to XII-a and initiated the coagulation cascade. Furthermore, the charged carboxymethyl CS also leads to the activation of factors XII and III, thereby initiating intrinsic coagulation. Such an approach aims to maximize the hemostatic effects of the hydrogel based on the hemostatic potential of both components of the matrix.

Cellulose is an abundant, renewable, and biodegradable polymer with high absorption and swelling capabilities. It has been utilized in various forms to develop hemostatic products, including gauze, sponges, hydrogels, films, and powders. Wei et al. employed a nonhomogeneous intermittent process to synthesize CS with a high degree of deacetylation. Subsequently, dialdehyde cellulose (DAC) was prepared by the interaction of cellulose nanocrystals with an oxidant [147]. Finally, a composite foam sponge was obtained by mixing and interacting CS and DAC via Schiff's base reaction. The prepared composite hemostatic sponge exhibited moderate strength and good elasticity. Compared with pristine CS and cellulose nanocrystals, it demonstrated rapid and enhanced hemostatic effects both in vitro and in vivo. This improved hemostatic foam dressing is promising for rapid hemostasis and wound management.

The availability of calcium at the injury site plays an important role in promoting prothrombin activation, stimulating platelets, and activating coagulation factors, such as VII, IX, and X, all of which are essential for enhancing hemostasis. Moreover, entanglement within the fibrous structure and mesh-like gel layers on the wound surface contribute to the effectiveness of blood clotting. Wang et al. prepared composite microspheres based on CA polysaccharides using a reverse emulsion method (Fig. 10c) [146]. The exceptional swelling properties enabled these microspheres to facilitate thrombus formation. Further experiments revealed that the microspheres increased platelet aggregation with an 80 % aggregation rate of red blood cells.

Owing to its unique molecular structure, HA plays a significant role in regulating vascular wall permeability, promoting cell proliferation, and enhancing wound healing. Notably, many inflammatory mediators that trigger coagulation are have also been demonstrated to stimulate HA synthesis. When combined with functionalized fibrinogen, HA facilitates the creation of a 3D matrix, enabling cell migration into the clot. Additionally, it interacts with thrombin, thereby modulating the inflammatory responses that are crucial for blood coagulation. Wang et al. prepared a novel polysaccharide-based hemostatic agent consisting of sodium trimetaphosphate-crosslinked starch and HA [148]. The composite hemostatic agent exhibited exceptional coagulation performance for superficial injuries and solid visceral and arterial wounds, comparable to commercial hemostatic agents. The inclusion of HA significantly improved the water-absorption capacity and hemostatic performance of the composite material.

### 5.2 Infection

Wound infections have long been a critical challenge in the clinical setting. They can exacerbate existing skin injuries, whereas the various external factors leading to wound formation can compromise or destroy the defense mechanisms of the skin. Furthermore, the loss of vascular nutrients established a local microenvironment that promotes bacterial colonization [[106], [107], [110]]. Wound sites are rich in plasma nutrients, attracting pathogenic microorganisms. The microorganisms multiply at the wound site and embed within the extracellular matrix. This leads to the formation of biofilms, which act as barriers to drug penetration, resulting in tissue infection and sustained inflammatory responses. The disruption of the normal tissue repair process ultimately leads to infected chronic wounds that fail to heal [149]. Hence, wound dressings must exhibit hemostatic properties and robust antibacterial activity to safeguard wounds against bacterial infections, thereby fulfilling clinical requirements. A direct and effective approach of achieving this is to incorporate antimicrobial agents into hydrogels or utilize materials with intrinsic antimicrobial properties.

**Fig. 11:**
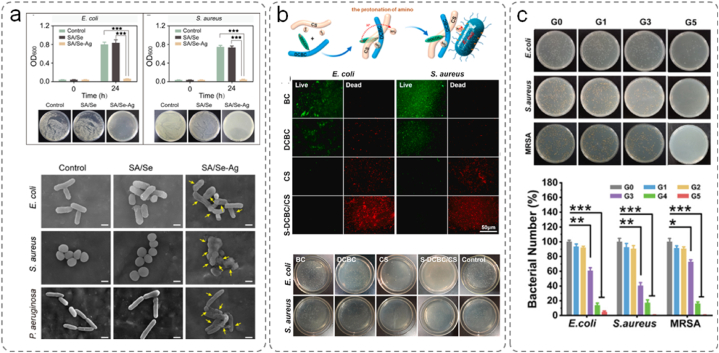
(a) Determination of bacterial growth and colony counts of *E*. *coli* and *S*. *aureus* by SA/Se–AgNP hydrogels. Bacterial membrane disruption observed using scanning electron microscopy [152]. Adapted reprinted with permission from Ref. [152], License Number: 5,795,360,481,217. *Copyright © 2020 Elsevier B.V. All rights reserved.* (b) S-DCBC/CS antimicrobial effect. Fluorescence micrographs of surface-cultured *E. coli* and *S. aureus*. Plate colony counting experiments [153]. Adapted reprinted with permission from Ref. [153], based on CC-BY–NC–ND License. *Copyright © 2022 The Authors.* (c) Sodium alginate oxide/carboxymethyl chitosan antimicrobial effect. Images of colonies on agar plates [154]. Adapted reprinted with permission from Ref. [154], License Number: 5,795,391,127,374. *Copyright © 2022 Wiley‐VCH GmbH.*

Silver has long been recognized as an effective antimicrobial agent [150]. Positively charged silver ions can interact with negatively charged thiol groups on bacterial membrane proteins via electrostatic forces, resulting in their incorporation into the bacterial membranes. The process leads to protein denaturation and, ultimately, bacterial apoptosis. Furthermore, the silver ions that bound as byproducts of bacterial apoptosis are reduced to silver atoms, providing silver with enduring antimicrobial properties. However, the strong oxidative nature of silver ions can cause indiscriminate tissue damage [151]. Silver nanoparticles are colloidal aggregates of silver. They only harm bacteria when they are oxidized into silver ions via enzymatic actions caused by microorganisms. Such a mechanism significantly reduces tissue irritation and extends the effectiveness of silver nanoparticles. Tao et al. synthesized an injectable hydrogel composed of sericin and sodium alginate (Fig. 11a) [152]. The calcium ions acted as crosslinkers, forming an interpenetrating network between sericin and sodium alginate. The loaded silver ions were reduced to silver nanoparticles by leveraging the phenolic hydroxyl groups of sericin. This hybrid hydrogel exhibited excellent injectability and moldability, whereas the released silver particles adhered to the bacterial cell membrane, disrupting their permeability. Simultaneously, the hydrogel generated ROS, causing bacterial death.

Meanwhile, antimicrobial peptides (AMPs) can bind to bacteria and cause damage by forming pores within the membrane or even dissociating bacteria through complex formation. Suo et al. used AMPs as crosslinkers to create a composite hydrogel (AMP-HA) through Schiff base reactions with HA [155]. AMP-HA triggered the release of AMP under acidic conditions. In a *Staphylococcus aureus*-infected wound model, the AMP-HA hydrogel exhibited significantly enhanced wound healing compared with AMP and the control group. The AMP and AMP-HA hydrogels demonstrated effective antimicrobial activity in the first five days of wound treatment.

Although antibiotic overuse can lead to bacterial resistance, antibiotics remain the preferred antimicrobial choice in clinical practice. However, current research has focused on the development of smart, nontoxic carriers that can provide controlled antibiotic release. When microorganisms adhere to wounds and reach a certain threshold of proliferation, the acidic byproducts of their metabolism can lower the local pH, leading to the disruption of specific chemical bonds such as borate esters and Schiff base bonds. Natural polysaccharides contain glycosidic bonds that can be easily oxidized by periodic acids, rendering them excellent sources of aldehydes. Consequently, a commonly employed approach involves the preparation of smart carriers by crosslinking oxidized polysaccharides via Schiff base reactions. Hu et al. developed a smart hydrogel composed of aminoglycoside antibiotics, pectinase, and oxidized dextran to treat localized biofilm-related infections [156]. When a bacterial infection occurs, the increased acidity triggers the release of pectinase and aminoglycoside antibiotics.

Compared with hydrogels supplemented with antimicrobial agents, hydrogels possessing inherent antimicrobial properties offer more versatile treatment options. CS is a polysaccharide exhibiting intrinsic antimicrobial characteristics owing to the numerous amino groups distributed along its chain, conferring it with strong cationic properties. CS targets negatively charged components such as peptidoglycans on bacterial membranes by adhering to microbial membranes via electrostatic interactions. This binding disrupts the osmotic balance of the microorganisms, leading to excessive swelling and apoptosis. Short CS chains can even infiltrate bacteria and bind to trace elements, thereby impeding microbial physiological activities and eradicating the bacteria. Xie et al. formed an aldehyde/carboxymethyl bacterial cellulose/CS composite material by self-crosslinking CS within a bacterial-cellulose network. The aldehyde groups reacted with the amino groups of CS via Schiff base reactions (Fig. 11b) [153]. The carboxyl groups in the bacterial cellulose and the amorphous distribution of CS molecular chains enhanced the antimicrobial performance of the composite material, effectively inhibiting bacterial proliferation and killing bacteria.

Although CS possesses antibacterial properties, its antimicrobial efficiency cannot compete with that of antibiotics or other antimicrobial agents. Consequently, various CS derivatives have been formulated to improve the antimicrobial performance of CS, demonstrating enhanced bactericidal capabilities compared to unmodified CS [157]. Wang et al. constructed a multifunctional PDA-modified core-shell nanofiber membrane with a growth factor-loaded core layer to promote hemostasis and wound healing [158]. The prepared nanofiber membrane achieved a bacteriostatic rate of 95 % against *Staphylococcus aureus* and 93 % against *Escherichia coli.*

External stimuli play a significant role in treating wound infections. The most common are light-based therapies, which can be categorized into photothermal therapy (PTT) and photodynamic therapy (PDT). In PTT, a photothermal material is used as a light source to eliminate pathogenic microorganisms during infection treatment. Copper ions are recognized for their antimicrobial and proangiogenic characteristics. Chung et al. designed CS/OA-based hydrogel carriers and loaded them with copper sulfide nanoparticles [159]. This hydrogel system exhibited excellent photothermal capabilities, eradicating bacteria almost completely under a near-infrared laser. Additionally, stimulation with copper ions significantly increased the proliferation of mouse wound tissue cells, collagen regeneration rate, and vascular density, thereby shortening the healing period.

Under light irradiation, PDT generates ROS that kills bacteria. Zhu et al. developed an oxygen-generating double-layered hydrogel that could visualize bacterial infection, provide oxygen to enhance antimicrobial photodynamic therapy and alleviate inflammation during diabetic wound healing (Fig. 11c) [154]. They combined oxygenated sodium alginate/carboxymethyl CS with a photosensitizing metal-organic framework and loaded agar and carboxymethyl CS with photosynthetic cyanobacteria that produce oxygen to improve the efficiency of antimicrobial PDT. The self-oxygenated double-layered hydrogels exhibited significant advantages for timely infection monitoring, synergistic treatment of refractory anaerobic bacterial wound infections, and tissue repair.

### 5.3 Inflammation

Inflammation, the primary response of the human immune system, is a physiological phenomenon that occurs in response to injury, infection, and stress. Various internal and external factors can disrupt the natural wound-healing process, with the inflammatory phase being particularly vulnerable [160]. In the early stages of inflammation, the injured tissues produce proinflammatory cytokines and chemokines. Neutrophils are essential for the removal of debris, engulfing invading bacteria, and producing ROS. At the wound site, macrophages are involved in angiogenesis, fibroblast proliferation, and ECM production, bridging the gap between the inflammatory and proliferative phases. Controlling inflammation is important for eliminating necrotic tissue, eradicating local bacteria, and facilitating wound healing. Maintaining a certain level of inflammation is essential for promoting wound healing while preventing interference with the process.

ROS are derivatives of O_2_, including hydroxyl radicals (•OH), superoxide anions (•O_2_^−^), peroxide (•O_2_^−2^), and hydroxyl ions (OH^−^). Elevated glucose levels or severe infections are unfavorable conditions within a wound, leading to prolonged infiltration of inflammatory cells, primarily neutrophils and macrophages, resulting in excessive production of harmful ROS [27,160]. Furthermore, such conditions limit the intrinsic antioxidant capacity of tissues. When the antioxidant capacity is insufficient, ROS can damage DNA, proteins, and cell membrane lipids, causing cell injury and apoptosis. Consequently, tissue damage triggers a cascade of inflammatory responses, inducing oxidative stress, which in turn sustains ongoing inflammatory infiltration and initiates a detrimental cycle that exacerbates the wound environment.

The integration of antioxidant components into multifunctional hydrogels through modification and aggregation can successfully mitigate the excess ROS in wounds, thereby promoting wound healing. Polysaccharides exert their antioxidant effects by directly or indirectly eliminating free radicals, enhancing the activity of antioxidant enzymes, and reducing the activity of oxidases. Additionally, polysaccharides can interact with various biomolecules, providing ample opportunities for designing hydrogels with specific beneficial properties for wound healing. For example, dextran is a naturally occurring polysaccharide produced by bacteria. It is known for its excellent moisture retention capabilities, rendering it suitable as a mild ROS scavenger to reduce platelet hyperactivation. Qiu et al. constructed hydrogels based on the amphoteric dextrans of carboxymethyl CS and sulfated CS [161]. Oh et al. prepared an oxidized alginate-gelatin hydrogel loaded with CS oligosaccharides and salicylic acid conjugates through graft polymerization [162].

Chemotactic factors constitute a subset of cytokines initially identified as substances that aid in recruiting white blood cells to the site of injury or infection, consequently releasing soluble factors [163]. However, persistent and excessive presence of chemotactic factors in wounds can lead to poor healing. Therefore, strategies for addressing excess proinflammatory chemotactic factors within wounds are required. Potential approaches targeting chemotactic factors include monoclonal antibodies that disrupt the distribution of chemotactic factors, small-molecule antagonists, and heparinoids. Qin et al. prepared a HA and gelatin composite hydrogel exhibiting anti-inflammatory activity [164]. The HA–gelatin hydrogel effectively reduced the expression of the proinflammatory chemotactic factor MCP-1 in the wound bed, thereby improving the healing outcomes in full-thickness wounds of diabetic mice.

Macrophages play a pivotal role in the early stages of wound healing by recognizing and clearing pathogens, cell debris, and apoptotic neutrophils, as well as facilitating the healing processes [45]. Macrophages are categorized into M1 and M2 macrophages based on their roles in wound healing. Analyzing the functions of these distinct macrophage types in wound healing is of utmost importance. M1 macrophages are responsible for producing reactive ROS, nitric oxide, interleukin-6, tumor Necrosis Factor alpha, and matrix metalloproteinase 9, which aid in the early recognition and clearance of pathogens, cell debris, and apoptotic neutrophils. In contrast, M2 macrophages contribute to the proliferation and remodeling phases of wound healing. They release significant levels of growth factors, while minimizing fibrosis and ECM remodeling. Adverse factors such as high blood sugar and bacterial infections hinder the conversion of M1 to M2 macrophages, resulting in the wound remaining in the inflammatory phase. This impairment delays epithelial regeneration, collagen deposition, and angiogenesis and obstructs the transition to the repair phase. Therefore, the promotion of sustained polarization of macrophages is essential.

**Fig. 12:**
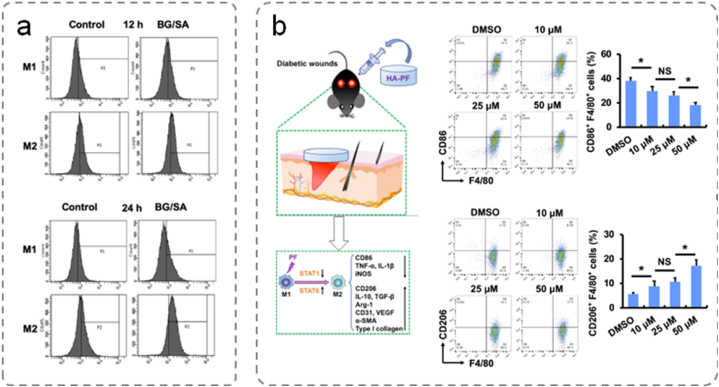
(a) Effects of the BG/SA hydrogel on polarization of RAW cells [165]. Adapted reprinted with permission from Ref. [165], License Number: 5,795,401,020,807. *Copyright © 2020 Elsevier Ltd. All rights reserved.* (b) Modulation of macrophages by a paeoniflorin-loaded hyaluronic acid-based hydrogel (HA-PF) promoting diabetic wound healing [166]. Adapted reprinted with permission from Ref. [166], License Number: 5,795,410,099,637. *Copyright © 2021 The Author(s). Published by Elsevier Ltd.*

Certain bioactive components can modulate the macrophage activity. Zhu et al. studied the impact of sodium alginate hydrogels loaded with bioactive glass (BG/SA) on macrophage behavior and their interactions with repair cells (Fig. 12a) [165]. The BG/SA hydrogel polarized macrophages toward the M2 phenotype both in vitro and in vivo. Yang et al. extracted paeoniflorin from *Paeonia lactiflora* and loaded it into a HA-based hydrogel (Fig. 12b) [166]. The hydrogel dressing significantly promoted the polarization of macrophages from the M1 to M2 phenotype.

### 5.4 Angiogenesis

**Fig. 13:**
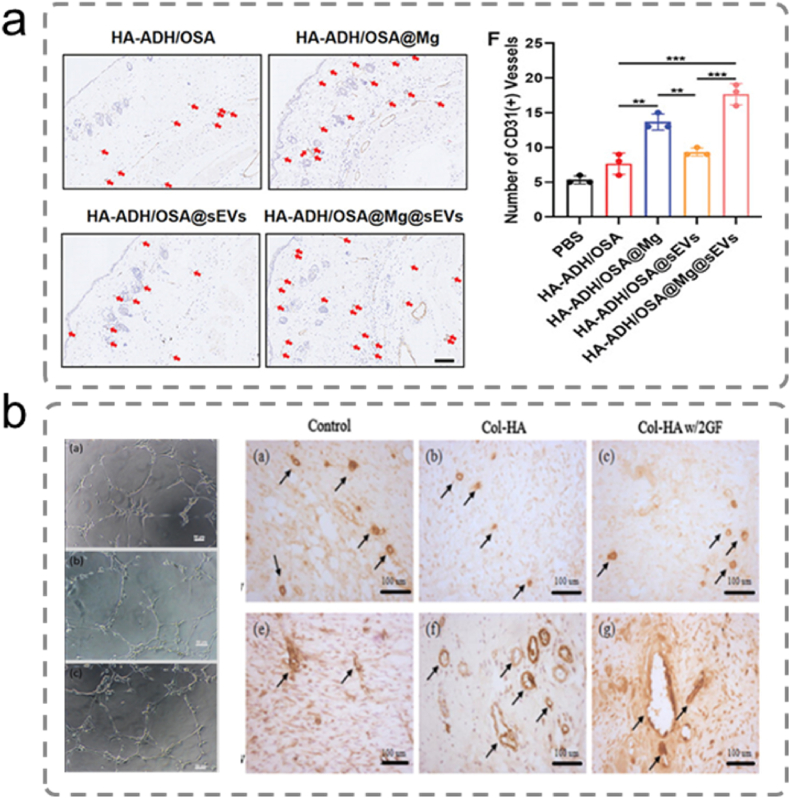
(a) HA-ADH/OSA @Mg hydrogel treatment of skin defects in rats. Skin tissue was collected from the diabetic mice in the five groups, and CD31 IHC assay was conducted to detect the newborn blood vessel [167]. Adapted reprinted with permission from Ref. [167], based on CC BY License. *Copyright © 2023 The Authors. Advanced Materials published by Wiley‐VCH GmbH.* (b) Growth factors directly embedded in HA nanofibers. Image of the capillary-like structures of HUVEC after 2 h of incubation in culture medium [168]. Adapted reprinted with permission from Ref. [168], License Number: 5,795,410,847,777. *Copyright © 2014 Acta Materialia Inc. Published by Elsevier Ltd. All rights reserved.*

Highly vascularized matrices ensure an ample supply of nutrients and oxygen to support the regeneration of new tissue at the wound site. Enhanced angiogenesis during skin regeneration accelerates wound healing and prevents necrosis. Numerous studies recognize the significance of angiogenesis in wound closure and have focused on this phase to accelerate the wound closure process. Xiong et al. designed an in situ injectable hydrogel for the delivery of engineered exosomes (EVs) as wound dressings (Fig. 13a) [167]. The HA-ADH/OSA@Mg@EVs hydrogel was formed by a facile one-step mixing of a HA-adipic dihydrazide (ADH)/magnesium (Mg) solution with an oxidized sodium alginate (OSA) solution and a suspension of engineered EVs. During the proliferative phase, enhanced angiogenesis was observed, owing to the synergistic effect of differentiated neural cells and the release of Mg^2+^.

Growth factors play pivotal roles in wound healing. Recent studies have focused on the use of growth factors to stimulate angiogenesis and improve closure in chronic wounds. Lai et al. created an electrospun nanofiber scaffold composed of collagen and HA, designed to release growth factors with different release kinetics (Fig. 13b) [168]. This structure accelerated wound closure and vascular maturation in the wound beds of diabetic rats.

Nitric oxide (NO) regulates endothelial cell growth, vascular tone, and angiogenesis. Following skin injury, NO levels significantly increase and gradually decrease as the wound healing process advances. Han et al. synthesized nanoparticles capable of releasing NO by encapsulating a mixture of CS, glucose, and sodium nitrite [169]. Within the matrix, sodium nitrite is reduced to NO, allowing the stable release of NO from the nanoparticles. In a mouse excisional skin wound model, local application of NO-releasing nanoparticles increased angiogenesis, as evidenced by the increased CD34 expression in the treatment area.

Statins exhibit proangiogenic properties, offering the potential to prevent ischemic injury and promote angiogenesis. Advanced drug delivery systems have been used to enhance the angiogenic capabilities of statins in the treatment of chronic wounds. Rezvanian et al. developed a simvastatin-freeze-dried chip using sodium carboxymethyl cellulose and methylcellulose and evaluated its potential in chronic wound therapy [170]. Using simvastatin as a model drug, the freeze-dried chips accelerated wound healing by promoting angiogenesis and lymphangiogenesis. The chip exhibited ideal wound dressing properties, including flexibility, hardness, sponginess, and adhesiveness, with sustained drug release characteristics necessary for wound healing and angiogenesis promotion.

## 6 Conclusions and perspectives

This article presents an overview of recent advancements in the preparation and processing of polysaccharide-based hydrogels and their derivatives, including CS, HA, alginate, and cellulose, along with their potential applications in wound repair. Polysaccharides are characterized by their natural abundance, low immunogenicity, cost-effectiveness, nontoxicity, and high water-absorbing capacity. Consequently, they are gaining popularity as materials for modern wound dressings. Various polysaccharide-derived hydrogels can be transformed into biomimetic platforms for use as wound dressings. These innovative wound dressings offer excellent hemostatic and wound-healing properties. Furthermore, the unique deformability and 3D porous network of polysaccharide hydrogels make them highly adaptable for processing into specific structures such as scaffolds, microneedles, and microspheres. This versatility renders them promising materials for tissue engineering applications.

Despite the recent advances in the use of polysaccharide-based hydrogels for wound healing, further improvement of these materials is required, addressing several critical issues that limit their applications. Currently, research is predominantly based on preclinical studies, which impose certain constraints on clinical translations. Consequently, future studies should include clinical data that prove and validate the applicability of polysaccharide-based hydrogels in wound healing. The development of innovative polysaccharides, including those derived from plants and algae, is of paramount significance for the fabrication of intelligent hydrogels. To create hydrogels for wound management, rigorous assessment of their quality from diverse perspectives is imperative.

Further studies investigating modifications of polysaccharide-based hydrogels or the development of alternative polysaccharide-based dressings are essential to achieve more precise control over the physicochemical properties of the gels, address a wider range of scenarios, and have a more pronounced biomodulatory effect on wounds. Compared to traditional hydrogel wound dressings, attempts have been made to integrate electronic components into hydrogels to monitor subtle changes in the wound site, allowing for a more accurate and comprehensive monitoring of the healing process. Recent research has explored the fabrication of microneedle-like hydrogels or the modification of their topological surface profile to better address the requirements of different types of wounds. Innovative manufacturing techniques must be developed to ensure stable production of biologically relevant materials associated with polysaccharides. Ultimately, with the advent of precision and personalized medicine, we anticipate that the development of polysaccharide-based hydrogel wound dressings will evolve toward more intelligent and portable ways. Polysaccharide-based hydrogels will remain an active area of research, encompassing both fundamental and practical investigations.

## Funding statement

This work was supported in part by Wenzhou science and technology project of China (No. Y20220935).

## 7 Data availability statement

No data were used for the research described in the article.

## Additional information

No additional information is available for this paper.

## CRediT authorship contribution statement

**Meifen Liu:** Conceptualization. **Jiman Jin:** Data curation. **Xiqiang Zhong:** Formal analysis, Data curation. **Liangle Liu:** Conceptualization. **Chengxuan Tang:** Writing – review & editing, Writing – original draft. **Limei Cai:** Writing – review & editing.

## Declaration of competing interest

The authors declare that they have no known competing financial interests or personal relationships that could have appeared to influence the work reported in this paper.
